# Supplementary material for: A 40-Year Cohort Study of Evolving Hypothalamic Dysfunction in Infants and Young Children (<3 years) with Optic Pathway Gliomas
Source: Cancers (Basel). 2022 Jan 31;14(3):747. doi: 10.3390/cancers14030747 (PMC8833541; doi:10.3390/cancers14030747)
Supplement: Supplementary file 1 [file cancers-14-00747-s001.zip › cancers-1496182-supplementary table S1.pdf]

cancers

MDPI

Supplementary Materials:

Supplementary Table S1. Demographic and endo-metabolic data in patients presenting with diencephalic syndrome vs those without diencephalic syndrome.

Factors and categories	Diencephalic Syndrome		*p* value
	Yes n=27	No n=63	
**AT DIAGNOSIS**			
**Gender male**	10 (37.0)	32 (50.8)	0.231
**Age (years)**	0.84 [0.50 – 1.90] (0.25-2.90)	2.00[1.20 – 2.60] (0.06-3.00)	**<0.0001**
**Age ≤1 year**	18 (66.7)	12 (19)	**<0.0001**
**NF1**	1 (3.7)	26 (41.3)	**<0.0001**
**Radiological Classification**			
MDC 1	0 (0)	19 (30.2)	**0.001**
MDC 2	4 (14.8)	21 (33.3)	0.072
MDC 3/4	23 (85.2)	23 (36.5)	**<0.0001**
**Hypothalamic involvement**	27 (100)	29 (46)	**<0.0001**
**Leptomeningeal Metastases**	9 (33.3)	2 (3.2)	**<0.0001**
**Visual symptoms at diagnosis**	12 (44.4)	29 (46)	0.890
**Raised intracranial pressure at diagnosis**	10 (37)	12 (19)	0.069
**AT LATEST FOLLOW UP**			
**Presence of endo-metabolic disorders**	23 (85.2)	29 (46)	**0.001**
**M-EMS(score 0-10)**	2 [1 – 5] (0 – 7)	0 [0 – 3] (0 – 7)	**0.002**
**APD**	21 (77.8)	26 (41.3)	**0.001**
**GHD***	16 (59.3)	21 (33.3)	**0.022**
**TSHD***	11 (40.7)	10 (15.9)	**0.011**
**ACTHD***	9 (33.3)	10 (15.9)	0.063
**CPP***	14 (51.9)	10 (15.9)	**<0.0001**
**GnD***	8 (29.6)	9 (14.3)	0.088
**PPD**	6 (22.2)	9 (14.3)	0.355
**CDI***	2 (7.4)	6 (9.5)	1.000
**SIADH***	4 (14.8)	6 (9.5)	0.479
**CSWS***	1 (3.7)	0 (0)	
**MD**	11 (40.7)	12 (19)	**0.031**
**Obesity***	11 (40.7)	12 (19)	**0.031**
**IGT/T2DM***	4 (14.8)	7 (11.1)	0.623
**Follow-up time (years)**	9.3 [3.6 – 15] (1.13-21.74)	9.9 [4 – 11.9] (0.52-25)	0.812
**Deceased**	5 (18.5)	4 (6.3)	0.121

Categorical variables are reported as number and percentage. Continuous nonparametric variables are presented as median [interquartile range] (range).

*Items of the Modified Endocrine Morbidity Score (M-EMS)

M-EMS: Modified Endocrine Morbidity Score; APD: Anterior Pituitary Dysfunction; GHD; Growth Hormone Deficiency; TSHD: TSH Deficiency; ACTHD: ACTH Deficiency; CPP: Central Precocious Puberty; GnD: Gonadotropin Deficiency; PPD: Posterior Pituitary Dysfunction; CDI: Central Diabetes Insipidus;

SIADH: Syndrome of Inappropriate Secretion for ADH; CSWS: Cerebral Salt Wasting Syndrome; IGT: Impaired Glucose Tolerance, T2DM: Type 2 Diabetes Mellitus.
