# Supplementary figures and images for: A 40-Year Cohort Study of Evolving Hypothalamic Dysfunction in Infants and Young Children (<3 years) with Optic Pathway Gliomas
Source: Cancers (Basel). 2022 Jan 31;14(3):747. doi: 10.3390/cancers14030747 (PMC8833541; doi:10.3390/cancers14030747)

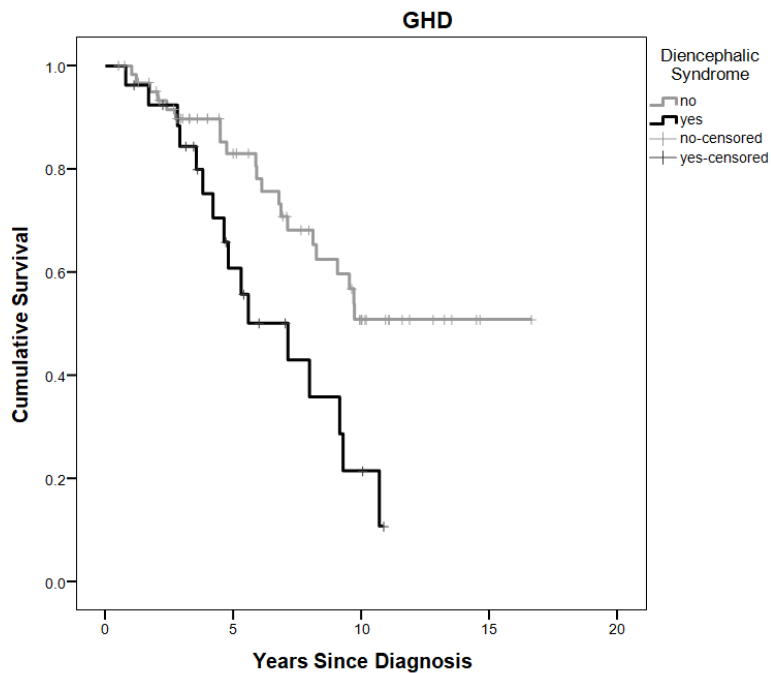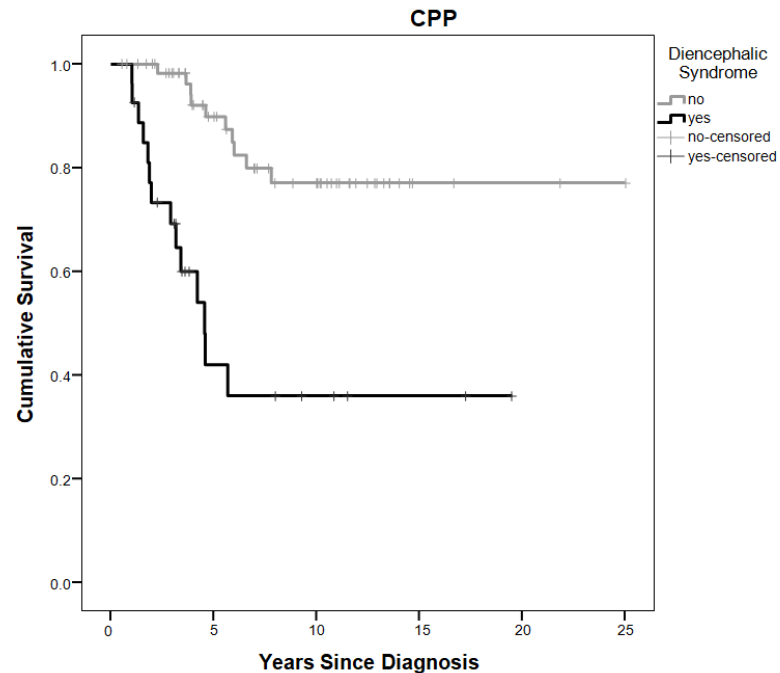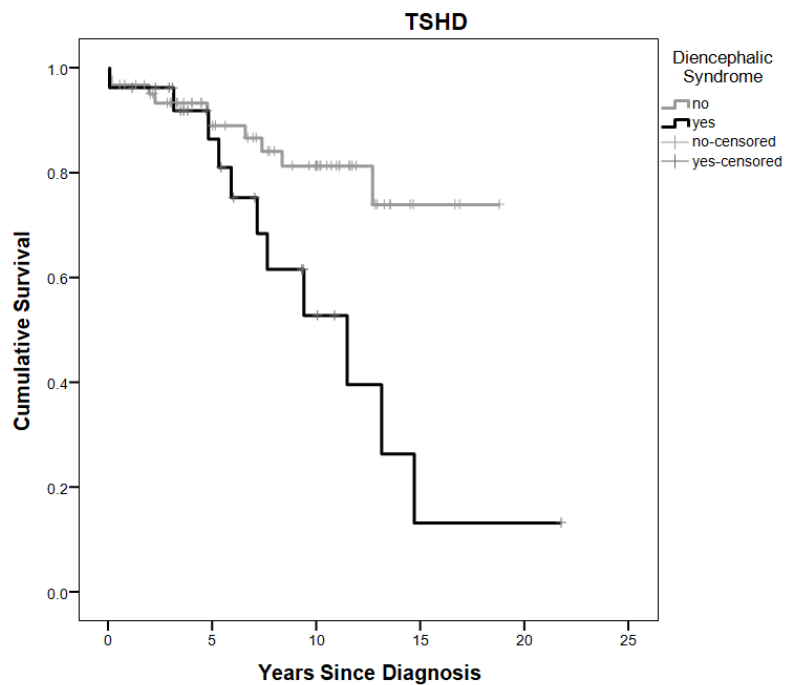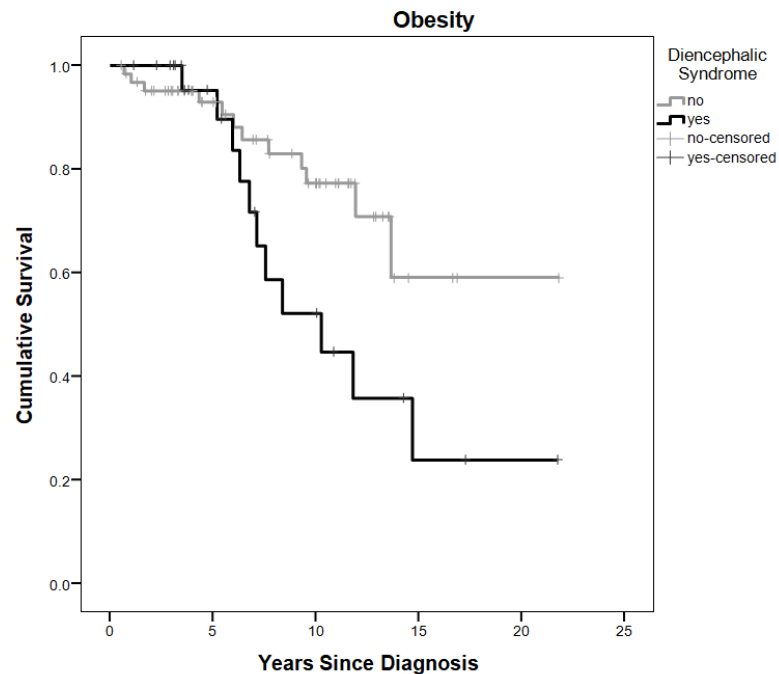

Supplement: Supplementary file 1 [file cancers-14-00747-s001.zip › Supplementary Figure 1.pdf]
